# Explainable Multimedia Feature Fusion for Medical Applications

**DOI:** 10.3390/jimaging8040104

**Published:** 2022-04-08

**Authors:** Stefan Wagenpfeil, Paul Mc Kevitt, Abbas Cheddad, Matthias Hemmje

**Affiliations:** 1 Faculty of Mathematics and Computer Science, University of Hagen, Universitätsstrasse 1, 58097 Hagen, Germany; matthias.hemmje@fernuni-hagen.de; 2 Academy for International Science & Research (AISR), Derry BT48 7JL, UK; p.mckevitt@aisr.org.uk; 3 Blekinge Institute of Technology, 371 79 Karlskrona, Sweden; abbas.cheddad@bth.se

**Keywords:** indexing, retrieval, explainability, semantic, multimedia, feature graph, graph code

## Abstract

Due to the exponential growth of medical information in the form of, e.g., text, images, Electrocardiograms (ECGs), X-rays, and multimedia, the management of a patient’s data has become a huge challenge. In particular, the extraction of features from various different formats and their representation in a homogeneous way are areas of interest in medical applications. Multimedia Information Retrieval (MMIR) frameworks, like the Generic Multimedia Analysis Framework (GMAF), can contribute to solving this problem, when adapted to special requirements and modalities of medical applications. In this paper, we demonstrate how typical multimedia processing techniques can be extended and adapted to medical applications and how these applications benefit from employing a Multimedia Feature Graph (MMFG) and specialized, efficient indexing structures in the form of Graph Codes. These Graph Codes are transformed to feature relevant Graph Codes by employing a modified Term Frequency Inverse Document Frequency (TFIDF) algorithm, which further supports value ranges and Boolean operations required in the medical context. On this basis, various metrics for the calculation of similarity, recommendations, and automated inferencing and reasoning can be applied supporting the field of diagnostics. Finally, the presentation of these new facilities in the form of explainability is introduced and demonstrated. Thus, in this paper, we show how Graph Codes contribute new querying options for diagnosis and how Explainable Graph Codes can help to readily understand medical multimedia formats.

## 1. Introduction and Motivation

In previous work on Multimedia Information Retrieval (MMIR), we demonstrated an efficient and effective approach for multimedia feature extraction, the semantic representation, annotation, and fusion of these features, and introduced information retrieval metrics including explainability of multimedia feature graphs [1,2,3]. Based on this MMIR research, further extensions and refinements are possible in the area of medical applications. A detailed R&D white paper on Artificial Intelligence for Hospitals, Healthcare & Humanity (AI4H3) [4] targets these extensions and refinements together with corresponding use cases. Furthermore, in the area of medical image processing and analysis, we have outlined and defined the process and detail of feature extraction on mammography images and their corresponding semantic representation [5,6,7]. In particular, the use cases given in [4] represent the motivation for the R&D approach herein.

**Figure 1:**
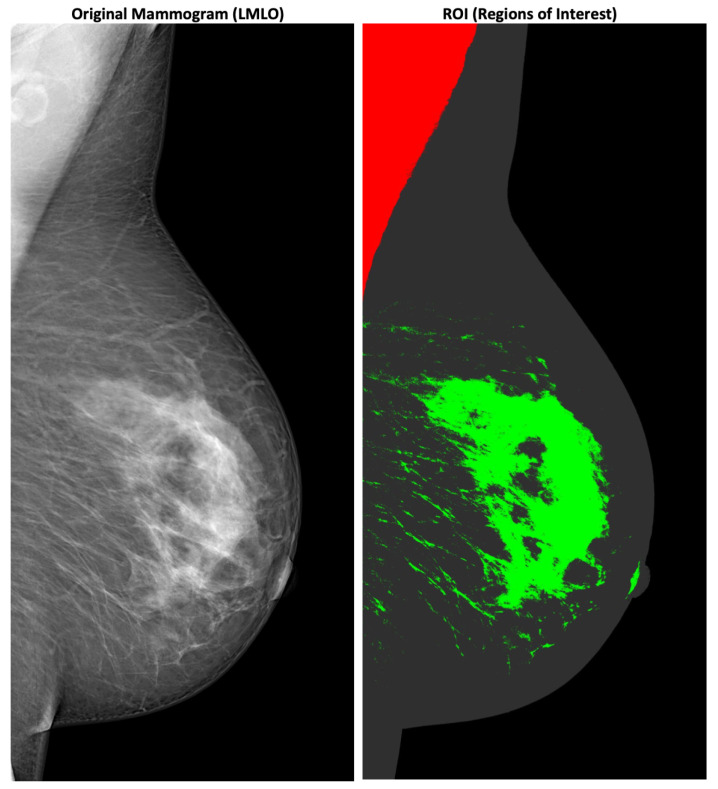
Example of a X-ray mammography with highlighted medical image features [7].

Medical image processing has been of interest since Wilhelm Röntgen discovered X-rays in 1895 [8]. The invention of Magnet Resonance Imaging (MRI) and Computer Tomography (CT) scans in the 1970s directly led to the production of digital images of a physical structure such as the human body [9,10]. Viewer applications such as the Digital Imaging and COmmunications in Medicine (DICOM) Viewer [11] provide a 3D experience for viewing medical images and extracting their relevant medical features, and hence offer innovative and accurate diagnostics. Medical image (and video) processing is important in modern healthcare. Modern technologies, e.g., Machine Learning (ML) or big data, are employed to detect, e.g., tumors or regions of interest automatically, which can produce highly annotated multimedia assets. Doctors’ letters are written in digital form and available for automated processing. Additionally, the quality and the Level of Detail (LOD) of technologies such as X-rays have increased significantly since their discovery in 1895. Analyzing X-ray images is increasingly supported by ML technologies. One example of this is the detection of tumors or regions of interest in X-ray mammograms to detect breast cancer [12]. As illustrated in Figure 1, medical images are typical examples of multimedia content including markups, overlays, descriptors, and detected features.

In the last 20 years, multimedia content has also become prominent in the area of assistive and preventive healthcare. This topic includes training of healthcare staff, education of patients, or patient rehabilitation programs, where multimedia content can be used to support diagnostics and other medical processes. The invention of multimedia devices, such as smartphones or tablets, has contributed significantly to digital workflows in hospitals, as doctors and nurses can access patient multimedia content data directly on devices and use such devices for medication, diagnostics, or documentation [13].

Furthermore, it must be considered that, in the medical domain, users have different levels of computing knowledge. Doctors and nurses having medical backgrounds may have limited knowledge in Multimedia Information Retrieval (MMIR) techniques. Hence, IR has to be presented in a human-understandable way. This becomes even more important when communication with patients should also be supported by MMIR techniques, as they might additionally have limited knowledge in the medical area. The explanation of a diagnosis, for example, has to be presented in a medically correct and human-understandable way to patients (see [4]) and with more medical detail and facts to nurses and doctors.

Therefore, in this paper, we address the overall problem that multimedia features derived in a medical context must be represented in an easily accessible and, in the same way, semantically understandable and explainable form. Two research questions correspond to this problem: (1) how can various medical multimedia features be integrated with each other? and (2) how can such medical feature information be represented semantically and made explainable for humans?

The structure of this paper follows the problem-solving approach of J. Nunamaker [14], which divides a problem into Observation, Theory Building, Implementation, and Experimentation phases, which are connected to each other. In Section 2 of this paper, the Observations are presented in the form of the current state of the art including a summary of the related technologies. Section 3 discusses the Theory Building (i.e., the modeling and design) of the proposed solution. In Section 4, implementation detail and evaluation are given, which correspond to the Implementation and Experimentation phases.

## 2. State of the Art and Related Work

This section provides an overview of current techniques and standards for multimedia information retrieval and medical applications and summarizes related work. A foundation is given based on existing approaches and the Graph Code concept is introduced as an efficient and effective indexing technique, as well as a summary of semantic analysis and intelligent information retrieval methods. Finally, in this section, the explainability of Multimedia Information Retrieval (MMIR) feature graphs is summarized.

### 2.1. Information Retrieval

**Figure 2:**
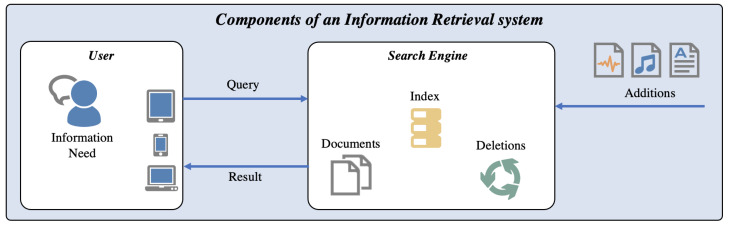
Visualization of a IR system and the corresponding components.

Following the terminology and introduction of Information Retrieval in [15], Figure 2 illustrates the uses cases, components, and processes that are part of an IR system. According to [15], the main component of an IR system is the Search Engine, which maintains the information database including the locations of encoded objects (often referred to as Documents). It also maintains the *Index*, which forms the data structure for searching and relevance ranking and provides a mapping between terms (i.e., words) and the locations of assets, in which these terms occur or which can be described by these terms. The process of extracting terms from documents is called *Indexing*. The process of finding the corresponding documents is called *Retrieval*. The search engine maintains the index and the document collection, and must consider the deletion and addition of documents. Both tasks can be system- or user-triggered (e.g., automated import vs. manual import). Typically, the User has an Information Need when working with a Search Engine. Therefore, the user can construct a Query, which is then processed in the search engine and will return Results.

To explain results selection, the Index is one of the most important components. Such an index can be represented as a B-tree (e.g., in relational databases) and simply provide sequential, ordered access to possible result elements. Indexes are extensive for more complex information. In [16], for example, multimedia index structures have been introduced, which are multidimensional, hierarchical, or recursive data structures to describe search-relevant features of multimedia assets. In many cases, these are stored in the form of feature graphs [17,18]. In previous work [2,3], we presented a unifying framework—the Generic Multimedia Analysis Framework (GMAF)—and data structure—the Multimedia Feature Graph (MMFG)—to represent such multimedia features in extended detail. The GMAF utilizes selected existing technologies as plugins to support various multimedia feature detection algorithms for text (e.g., social media posts, descriptions, tag lines) [19,20,21], images (especially object detection and spatial relationships including the use of machine learning) [18,19,20,22,23], audio (transcribed to text) [22,24], and video, including metadata [25] and detected features [26,27].

In modern IR applications, the terms *Weighting*, *Filtering*, and *Profiling* are employed to describe the need to reduce the list of possible results by applying additional criteria for retrieval [28]. Weighting describes the (manual or automatic) task of assigning relevance values to specific information types to increase or decrease their effect on retrieval. Filtering means that certain collection elements are excluded from IR processing. Such filters can be defined by users, administrators, or dynamically in terms of Relevance Feedback. The reusable combination of Weighting and Filtering for distinct application domains is then called Application Profiling, which means that such a set of weights and filters is applied to any application of a certain domain to produce uniform and comparable IR results [18,28].

Another key topic in IR is the maintenance and classification of valid and invalid information. This can be achieved by Truth Maintenance Systems [28], which in many cases employ nonmonotonic reasoning [29] to calculate true or false information. The information base can be represented by a so-called Default Theory [30] and can be defined according to Poole [30,31]. Such a Default Theory $T_{Poole}=\left(F,D\right)$ is described by the set F of facts (i.e., justified beliefs) and the set D of hypotheses (i.e., possible assumptions). The overall goal is to increase the number of elements in F by applying rules or calculations to the elements in D (i.e., building Knowledge Extensions). This is achieved by defining a measure for the Knowledge Validity KV of contained information. Based on this measure, reasoning and inferencing can be introduced to MMIR applications. However, the calculation of such a Knowledge Validity needs to be defined. In addition to valid and invalid information, in some IR systems, the term *Aboutness* is also used, which describes the overall topic domain of the MMIR application [32]. In the medical context, this might be, e.g., mammography, orthopedics, and hematology.

### 2.2. Multimedia Features and Multimedia Feature Graphs

The GMAF provides a flexible and extendable plugin architecture for the processing of various multimedia asset types. Such a plugin is based on a simple API and can be written to extract features from a special image, video, text, or audio format. These plugins contribute their extracted features to the MMFG, where they can be further processed. When the LOD of the given assets increases, the number of MMFG elements (i.e., nodes and edges) also increases. To provide a fast and effective indexing solution for this, we introduced Graph Codes (see Section 2.3). Further extensions of MMFGs lead to a semantic annotation (and to Semantic Multimedia Feature Graphs (SMMFGs)) and even to Explainable SMMFGs (ESMMFGs), which are outlined in Section 3. However, even with these extensions and annotations, the pure graph-based structure of an MMFG remains. As we showed in [2], this graph-based structure leads to exponential processing time of some MMIR algorithms. Particularly, for medical applications, it is important to integrate a high number of features from various sources into such an MMFG and to increase the LOD to the best possible extent. Therefore, exponential processing times are not acceptable and need to be mitigated by the introduction of an improved processing model. This is outlined in the next subsection.

### 2.3. Graph Codes

**Figure 3:**
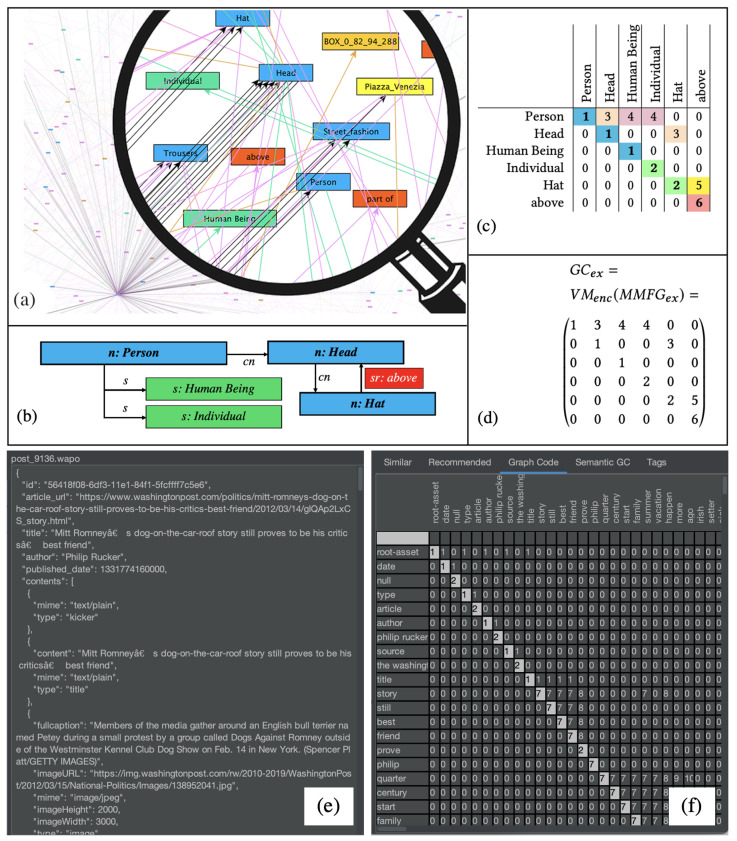
Multimedia features represented as a Graph Code index (**a**–**d**), example of a Graph Code index and its matrix visualization for a text document (**e**,**f**).

Graph Codes [2,33] are a 2D projection of a multimedia feature graph based on adjacency matrix operations [34], where the feature vocabulary terms of a feature graph are presented by row and column indices, and matrix positions represent relationships between these features. It has been demonstrated that Graph Codes are efficient for similarity and recommendation searches, as they basically use linear growing matrix operations instead of exponentially growing graph-traversal-based algorithms. In Figure 3, a visualization of an MMFG (see Figure 3a), a simple example for further illustration (Figure 3b), the corresponding Graph Code in a table representation (Figure 3c), and the Graph Code matrix $GC_{ex}$ (3d) are illustrated. A “real-world” scenario of such a Graph Code is shown in Figure 3e,f.

Graph Codes contain a dictionary $dict_{GC}$ of feature vocabulary terms FVT (i.e., the row and column descriptions) and represent the relationships between dictionary terms as value of a matrix field $m_{i,j}$. As Graph Codes represent multidimensional MMIR features, a similarity metric triple $M_{GC}=\left(M_{F},M_{FR},M_{RT}\right)$ has been defined, containing a feature-metric $M_{F}$ based on the vocabulary, a feature-relationship-metric $M_{FR}$ based on the possible relationships, and a feature-relationship-type-metric $M_{RT}$ based on the actual relationship types. This similarity metric can be applied for the calculation of MMIR results. In [1], we already introduced Semantic Graph Codes (SGCs), which contain additional semantic structures by an annotation with semantic systems such as RDF, RDFS, ontologies, or knowledge organization systems [35,36,37]. This semantic information can be utilized to bridge the semantic gap between the technical representation of MMIR features and their human-understandable meaning. This can be supported by the introduction of grammars and is outlined in the next subsection.

### 2.4. Explainability of MMFGs Supported by Natural Language Grammars

In order to support explainability, the automated production of natural language text and associated grammars [38] can be employed. On this basis, an algorithmic implementation can distinguish between valid and invalid statements of a given formal or natural language. According to [38], a grammar G=(V,T,P,S) for a language *L* is defined by the tuple of vocabulary terms *V*, the list of terminal symbols *T*, which terminate valid sentences of *L*, and production rules *P*, which describe valid combinations of vocabulary terms and a set of starting symbols *S* for sentences of *L*. In [39], PS-Grammars are employed as a specialized form to generate language terms by production rules, in which the left side of the rule is replaced by the right side. If, for example, α→β is a production rule in *P*, and ϕ, ρ are literals in *V*, then ϕαρ→ϕβρ is a direct replacement.

- $V_{en}=\left\{S_{en},NP,VP,V,N,DET,PR\right\}$ represents the variables of the grammar;
- $T_{en}=\left\{the,hat,is,above,head\right\}$ is the set of terminal symbols;
- $P_{en}$ is the set of production rules for this grammar and can be defined as follows:
  (1) $$\begin{array}{c} P_{en}=\{S_{en}\to NP\;VP, \end{array}$$
  (2) $$\begin{array}{c} VP\to V\;PP, \end{array}$$
  (3) $$\begin{array}{c} NP\to DET\;N, \end{array}$$
  (4) $$\begin{array}{c} PP\to PR\;NP\}. \end{array}$$

The production rules of PS-Grammars can be visualized in the form of so-called PS-trees [39]. Further details on PS-Grammars are given in our previous work [1]. By applying these production rules, both the construction and the analysis of well-formed sentences can be improved. In the next section, a formal grammar for Graph Codes and the corresponding concepts is defined and forms the basis for explainable MMIR.

**Figure 4:**
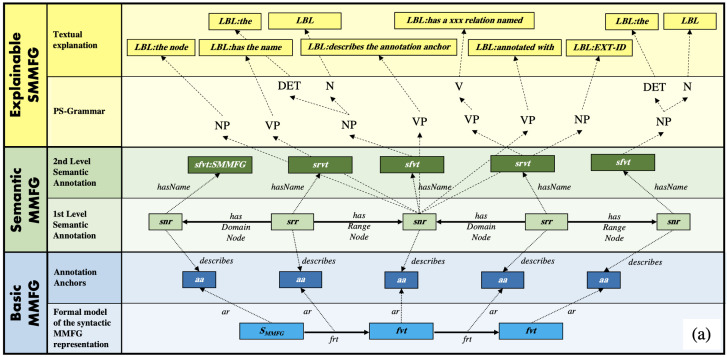

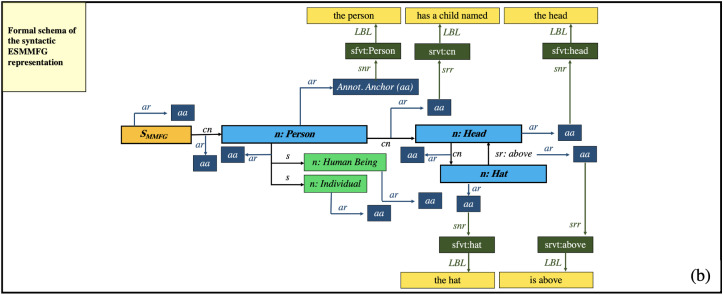
ESMMFG overview: (**a**) formal model of syntactic ESMMFG representation, (**b**) formal schema of syntactic ESMMFG representation.

In [1], we showed that MMFGs can be explained in a human-understandable natural language manner by introducing semantic annotation anchors, which not only represent the vocabulary terms of an MMFG, but also the syntactic structures, with which they are connected to each other (see Figure 4). This fully automated explainability is achieved by formally modeling the syntactic representation of MMFG elements and annotating them with semantic concepts, which can then be transferred by production rules into a PS-Tree for natural language processing.

The required concepts and techniques for MMIR processing have been introduced. However, when entering the area of medical applications, some further prerequisites are required. This is outlined in the next subsection.

### 2.5. Medical Multimedia Information Retrieval Applications

One very general difference to normal MMIR applications in the medical area is that typically any multimedia object comes with a textual description in the form of a doctor’s letter. Hence, the processing of this textual information is highly important during feature extraction. In addition, various medical formats have been established. A short summary of the most common formats according to [40] can be given as follows:

- The Analyze-format was created at the end of 1980s and was the “de facto” standard for medical imaging post-processing. It has been designed for multidimensional data, which enables storing 3D or 4D data in one file. The format consists of two binary files, one .img file containing the raw image data, and an .hdr header file containing metadata. However, the header size is fixed and can only contain 348 bytes.
- The Neuroimaging Informatics Technology Initiative (NIfTI) format has its origin in the early 2000s and was intended to revise the *Analyze format* with the intention to create a format for neuroimaging maintaining the advantages of the *Analyze format*, but solving the weaknesses. NIfTI can in fact be thought of as a revised *Analyze format* and has rapidly replaced Analyze in neuroimaging research. The header size has been extended, and an additional .nii file is produced, in which header and pixel data of images are merged.
- MINC was developed at the Montreal Neurological Institute (MNI) starting from 1992 to provide a flexible data format for medical imaging. The first version of the MINC format (MINC1) was based on the standard Network Common Data Format (NetCDF). Subsequently, to overcame the limit of supporting large data files and to provide other new features, the MINC development team chose to switch from NetCDF to Hierarchical Data Format version 5 (HDF5). This new release, not compatible with the previous one, was called MINC2.
- The DICOM standard was established by the American College of Radiology and the National Electric Manufacturers Association in 1993. Today, the DICOM standard is the backbone of every medical imaging department. The added value of its adoption in terms of access, exchange, and usability of diagnostic medical images is, in general, huge. DICOM is not only a file format but also a network communication protocol, and although the two aspects cannot be completely separated, here, we will discuss only DICOM as a file format. The innovation of the DICOM format was that metadata and pixel data are merged in a unique file, and the DICOM header, in addition to the information about the image matrix, contains the most complete description of the entire procedure used to generate the image ever conceived in terms of acquisition protocol and scanning parameters. The header also contains patient information such as name, gender, age, weight, and height.

**Figure 5:**
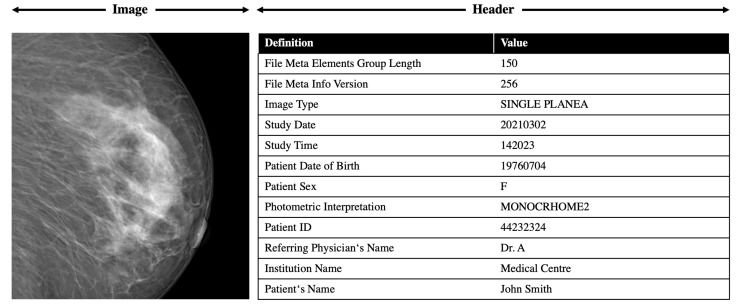
Exemplary snippet of the DICOM feature description.

All these formats can produce features and feature descriptions in a machine readable format. An exemplary snippet of the DICOM feature extraction is shown in Figure 5.

One further and key objective to any application in the medical area is data protection. Security and privacy have to be guaranteed for any kind of medical data. The GMAF has built-in support for securing information. However, this topic is beyond the scope of this paper.

### 2.6. Discussion and Remaining Challenges

In this section, we outline that the current state of the art covers many aspects which are relevant for building MMIR applications in the medical application domain. However, there are a number of remaining challenges. In the area of Information Retrieval, one major open challenge is how medical features can be related to each other and how queries in such a context can be processed. Furthermore, the presentation of retrieval results needs additional explanations and transparency in order to prove its correctness and accuracy. The Multimedia Feature Graph (MMFG) as such is a supportive and flexible data structure. However, the remaining challenge in this area is to determine which MMIR features are of what relevance in the medical context and from which assets and in which form they can be extracted. The current state of the art in the area of Graph Codes shows that highly efficient and effective MMIR can be built based on qualitative discrete relationships between feature vocabulary terms. However, in the medical context, further feature relationships, pre-processing steps (e.g., filtering or weighting), and a quantitative extension are required to represent discrete medical values. Furthermore, a semantic representation of the MMIR indexes is required to determine relevant information for a specific context. Finally, in terms of Explainability, different roles have to be supported, as the language and terminology used for conversations with family members of a patient should be completely different to conversations between doctors. These open challenges will be closed based on existing Medical MMIR formats and applications and addressed in the remainder of this paper.

As a basis for our modeling, we employ approved and reliable Multimedia Information Retrieval (MMIR) techniques and extend or adapt them to meet the requirements of medical applications and data. This model is based on the Generic Multimedia Analysis Framework (GMAF), the Multimedia Feature Graph (MMFG), and the corresponding Graph Codes as indexing structures and defined in the next section.

## 3. Modeling and Design

In this section, the building blocks of the proposed research and development approach are introduced. This modeling follows the methodology of N. Draper’s “user-centered system design” [41], which places the user at the center of modeling. Applied to the medical context, this means that doctors, nurses, and patients are typical actors for MMIR use cases. The previously introduced building blocks GMAF, Graph Codes, MMFG, and the corresponding concepts will be employed as a basis for the modeling discussed in this section. In this and the following sections, images are often employed as examples for medical MMIR feature processing. However, as shown in previous work [1,2], this modeling can be applied to any other multimedia asset type. This includes motion captures, surveillance stream data, pixel tagging and tracing methods, and other already existing feature extraction methods.

While in typical multimedia applications each asset is represented individually by its own MMFG and its own Graph Code, in medical applications we regard the patient quite literally as a multimedia object. This means that multiple multimedia assets can contribute information to the patient’s information base. Whenever a new X-ray image of the patient is available, whenever new blood parameters have been measured, whenever a new doctor’s letter is written, all these single multimedia objects and their corresponding Graph Codes are part of the patient as a multimedia object. This further means that a patient’s Graph Code $GC_{P}$ can be regarded as the union of all (e.g., *n*) available information from single multimedia assets $GC_{i}$:

(5) $$\begin{array}{c} GC_{P}=\overset{n}{\underset{i=1}{⋃}}GC_{i}. \end{array}$$

However, during the time, the information base changes. New X-ray images are taken, new blood parameters are measured, and further doctor’s letters are written. As for diagnosis, historical data are of high importance, each new contribution of an asset to $GC_{P}$ is represented by a timed Graph Code, which contains only information until a specific (or current) date. Previous versions of $GC_{P}$ will be retained. The latest Graph Code representing the most current information of a patient will be called $GC_{P}\left(t\right)$, the previous versions will be called $GC_{P}\left(t-1\right),\ldots ,GC_{P}\left(t-n\right),GC_{P}\left(0\right)$. This also means that $GC_{P}\left(t\right)$ is the union of all previous versions of patient information. The introduction of such a timeline can be also the basis for ML-based prediction and analytics algorithms. It is also helpful, if at the beginning of such a timeline, information of normal organ systems is also collected. In this case, the detection of deceased systems and the comparison of features to the normal state can be improved significantly.

(6) $$\begin{array}{c} GC_{P}\left(t\right)=\overset{t}{\underset{i=0}{⋃}}GC_{P}\left(i\right). \end{array}$$

This modeling also prepares the option to compare two different timed versions $GC_{P}\left(t_{1}\right),GC_{P}\left(t_{2}\right)$ of a complete set of patient’s information. The difference $GC_{P-Diff}$ between these versions can then be calculated by the subtraction (i.e., the disjunction) of the Graph Codes:

(7) $$\begin{array}{c} GC_{P-Diff}\left(GC_{P}\left(t_{1}\right),GC_{P}\left(t_{2}\right)\right)= \end{array}$$

(8) $$\begin{array}{c} GC_{P}\left(t_{1}\right)-GC_{P}\left(t_{2}\right)= \end{array}$$

(9) $$\begin{array}{c} \left(GC_{P}\left(t_{1}\right)\cup GC_{P}\left(t_{2}\right)\right)\cap GC_{P}\left(t_{1}\right). \end{array}$$

As $GC_{P}\left(t\right)$ contains any information from any multimedia object related to the patient at a specific time, this difference calculation between two timed Graph Codes can be extremely supportive in the area of diagnosis. Thus, answers to questions such as, “what happened between January and July?”, can be calculated based on $GC_{P-Diff}$. The presentation of such results in a human-understandable way is discussed in Section 3.4. However, before this, some further modeling, prerequisites, and extensions to the current Graph Codes are introduced.

As Graph Codes are constructed by the GMAF, various plugins for the medical context are required to parse and interpret medical features and to represent them as MMFGs and the corresponding Graph Codes. Examples for these plugins are given in Section 2 and are illustrated in more detail in Section 4. For the further modeling, it is important to outline that compared to typical MMIR applications, there are certain differences in the area of medical or diagnostic applications. In the following subsections, some refinements and extensions for Graph Codes are introduced.

### 3.1. Extension of Feature Description Attributes with Value Ranges

For medical applications, it is required to work on quantitative and continuous value ranges in addition to the already existing qualitative and discrete values for feature relationships. This supports further and more flexible querying. Typical examples for this are the measurement of blood pressure and the general metadata of a patient (e.g., height and weight), but also specific feature values detected by X-ray applications (e.g., the diameter of a tumor).

**Example** **1.** *To further illustrate the effect on Graph Codes, the detection of faces and their biometrical landmarks is employed as an example, where each detected face is represented by a set of such landmarks (e.g., position of left eye, position of nose) and their distance vectors (e.g., distance between left eye and right eye). Table 1 shows a Graph Code with the landmark “Eye Distance” for two faces. Until now, the values of landmarks would be represented as detected features in a Graph Code, which then would result in a vocabulary term representing the discrete value of these landmarks.*

However, this representation leads to potentially unlimited elements in a Graph Code’s dictionary. Therefore, for representation of discrete mathematical values, a special relationship type Discrete (*D*) is introduced, which directly represents the mathematical value instead of a relationship type.

**Example** **2.** *In the case of the Graph Code of Example 1, the corresponding Graph Code with discrete values is shown in Table 2.*

When constructing Graph Code Queries, these value ranges can be defined by placing a Discrete relationship type into the Query Graph Code, which contains a range of possible numeric values, e.g., D(27.10–27.25), indicating a minimum or equal range value of 27.10 and a maximum or equal range value of 27.25. Whenever the Graph Code Metric $M_{RT}$ is applied to a Discrete relationship type, it will not calculate. If the values in the corresponding fields are equal, it will calculate, if the value of the Graph Code’s matrix cell is within the range defined by the Discrete relationship. Graph Code Value Ranges also represent the basis for the IN-operation, which empowers the user or applications to construct queries such as where eye distance IN (27.10, 27.25). The EQUALS-operation can also be implemented on this basis to construct queries such as where age EQUALS 21. With the help of these operations, a bridge to existing relational databases can also be designed, which constructs Graph Codes on the basis of SQL statements. In this case, the columns of a SQL result set are mapped to the Graph Code Dictionary and the values of each result row are placed as discrete relationships into the corresponding Graph Code. This mechanism can be utilized to fuse existing content of relational databases into multimedia collections.

**Example** **3.** *Table 3 illustrates how Graph Codes can be constructed based on the exemplary SQL query select p.firstname, p.lastname, a.street, a.zipcode from patient p, address a where a.person-id = p.id:*

Of course, storing each row in a single Graph Code is inefficient compared to relational database technologies. However, this approach gives access to the full potential of Graph Codes and the corresponding metrics, and thus enriches the information stored in $GC_{P}$ or $GC_{P}\left(t\right)$. However, attaching relational databases to Graph Codes will produce significant additional data, which must be processed according to its overall relevance.

### 3.2. Feature Relevance Weighting Supporting Application Profiling

In medical applications, a $GC_{P}\left(t\right)$ contains any available information related to any multimedia object (i.e., text, audio, image, video, including X-ray, and ECG) and thus becomes extensive. Although Graph Codes provide outstanding MMIR performance, an optimization in the form of relevance weighting and filtering must be introduced. If MMIR collections are volatile, i.e., new and/or different assets are added to the collection frequently, solutions for the automated calculation of such weighted filters, for the modeling of topics, and for the detection of relevant feature vocabulary terms must be employed. Such systems can be called Calculated Simple Knowledge Organization Systems (CSKOSs) and are typically based on a Relevance Metric. While the already introduced existing Graph Code Metric $M_{GC}=\left(M_{F},M_{FR},M_{RT}\right)$ is designed to represent the similarity of two Graph Codes, for medical applications, further considerations in terms of Relevance must be made.

In the following subsections, the definitions and examples of metrics demonstrate this requirement. The metrics are introduced “top down” in order to follow a logical and easy-to-understand sequence, starting from the most general relevance definition (i.e., the Aboutness in Section 3.2.1), the collection relevance (i.e., the Feature Relevance in Section 3.2.2), and ending at the Graph Code relevance (i.e., the Feature Discrimination in Section 3.2.3). In Section 3.2.4, the calculation model for these metrics is then defined by employing a “bottom up” approach, which ensures a formal mathematical foundation and leads to the definition of Feature Relevant Graph Codes in Section 3.2.5.

#### 3.2.1. Aboutness Metric

As $GC_{P}\left(t\right)$ contains any available information related to any multimedia object of a patient *P* at a certain time *t*, mammography images and X-ray images of broken legs are, for example, available in the same collection. However, if the medical application is breast cancer detection, broken leg images are not relevant for the application. This Aboutness must be determined to remove irrelevant MMIR assets from the collection, and thus increase the relevance of the remaining assets for the medical application domain. As a measure for this Aboutness, we define the metric $M_{ABT}$ as the Aboutness Relevance metric to represent the collection’s topical domain. It can be employed to determine if an MMIR asset is in general relevant for a specific application domain.

**Example** **4.** *A patient visits an oncologist and brings, e.g., all his documentation and images. Then, the Aboutness metric would automatically remove anything that does not belong to the area of Oncology.*

#### 3.2.2. Feature Relevance Metric

Within the elements of a collection, various features are detected. The textual analysis of doctors’ letters, for example, will produce a varying number of vocabulary terms, some of which might also exist in DICOM images, or in the description of X-ray images. However, only some of these terms are relevant for a medical MMIR application. To describe this feature relevance, we define the metric $M_{REL}$ as the Feature Relevance metric and describe how important a certain feature vocabulary term is for an MMIR collection. This can also be regarded as a discriminator, which eliminates irrelevant feature vocabulary terms from a collection.

**Example** **5.** *The term “tissue density” may be a relevant MMIR feature when it has distinguishing power, i.e., it represents MMIR features that are relevant for querying and able to distinguish possible result elements. This further means that the value of such a “tissue density” is of importance for the application user. The feature vocabulary term “software version” may also occur in many Graph Codes. However, it might not be of importance for the MMIR application, as it is not expected to have distinguishing power or importance for the user.*

#### 3.2.3. Feature Discrimination Metric

Finally, the actual value of a feature vocabulary term can have discriminating power. This means that the feature is important within a single asset. For example, the feature “tissue density” might be irrelevant for MMIR processing, if its value is within the “normal” range of ”tissue densities“. If the value is higher than normal, this feature becomes discriminative, and increases the relevance of a feature’s value of an asset within the MMIR applications. The Discriminating Power of a feature is defined by the metric $M_{DIS}$ and measures how important a certain feature vocabulary term is within a collection element.

**Example** **6.** *An example of this metric would be a retrospective cohort study of certain patients, where distinct feature values change over time. In addition, the comparison of a certain feature value to a collection of Graph Codes of other patients can be based on such a feature Discrimination.*

#### 3.2.4. Calculation Model for These Metrics

The metrics defined above are now integrated in the Graph Code model. As a calculation basis for the modeling of these metrics, we selected a statistical method, the Term Frequency Inverse Document Frequency (TFIDF) measure [42]. Other approaches (e.g., machine learning) are also valid measures for determining the relevance of a feature vocabulary term within a collection [28]. However, it has been proven that statistical methods such as the TFIDF can deliver highly efficient and effective results [43,44] for text-based topic modeling. Hence, in the following, an adaptation of this algorithm for Graph Codes is discussed, which is based on Semantic Graph Codes, as relevance calculations typically require further semantic information. With this, a more qualified decision can be made as to which general topic a certain vocabulary term belongs (e.g., the term “Jaguar” can belong to the topic of animals, but also to the topic of the automotive industry). Hence, in the following, we employ SGCs instead of GCs, as a semantic extension supports the calculation of relevance.

First of all, the metric $M_{DIS}$ for feature discrimination is now defined as the difference in the number of nonzero Graph Code fields for two feature vocabulary terms $vt_{i}$, $vt_{j}$ of a given Graph Code or Semantic Graph Code. The greater this difference is, the more relevant (i.e., discriminative) the corresponding feature vocabulary term is. If, for example, a term has many relationships, it becomes discriminative for this Graph Code. $M_{DIS}$ of two feature vocabulary terms $vt_{i}$, $vt_{j}$ thus can be defined as the number of all nonzero matrix fields m(i,j):

(10) $$\begin{array}{c} M_{DIS}\left(vt_{i},vt_{j}\right)=\sum_{k=0}^{n}|m\left(i,k\right)\neq 0|-\sum_{k=0}^{n}\left|m\left(j,k\right)\neq 0\right|. \end{array}$$

Secondly, the TFIDF measure is adapted for *Graph Codes*. In general, TFIDF measures, how representative a term *t* is for a single document *d* (i.e., the term frequency #(t,d)), and in how many documents this term occurs (i.e., the document frequency #D(t)) can be found by calculating:

(11) $$\begin{array}{c} TFIDF\left(t,d\right)=\#\left(t,d\right)\cdot log\frac{\left|D\right|}{\#D(t)}. \end{array}$$

To apply this measure for Semantic Graph Codes (*SGC*), the following modifications and mappings are made. Basically, SGC vocabulary terms $vt_{i}$ of Semantic Graph Codes correspond to the TFIDF terms *t*, a Semantic Graph Code corresponds to a TFIDF document *d*, and thus, the collection of Semantic Graph Codes $SGC_{Coll}$ corresponds to the TFIDF documents *D*. Applying this, for each term, the document frequency can be calculated according to the general TFIDF function. However, calculating the term frequency #(t,d) based on a SGC is not possible, as a SGC’s dictionary is based on unique items. Hence, we employ the metric $M_{DIS}$ as a (even better) measure for the importance of terms $vt_{i},vt_{j}$ of SGCs:

(12) $$\begin{array}{c} \forall vt_{i}\in SGC,\;\forall vt_{j}\in SGC_{Coll}: \end{array}$$

(13) $$\begin{array}{c} TFIDF\left(vt_{i},SGC\right)=M_{DIS}\left(vt_{i},vt_{j}\right)\cdot log\frac{|SGC_{Coll}|}{M_{DIS}\left(vt_{i},vt_{j}\right)}. \end{array}$$

Now, the metric $M_{REL}$ representing the Feature Relevance can be defined as the difference in the TFIDF measure of two feature vocabulary terms according to the overall collection:

(14) $$\begin{array}{c} \forall vt_{i},vt_{j}\in SGC_{Coll}: \end{array}$$

(15) $$\begin{array}{c} M_{REL}\left(vt_{i},vt_{j}\right)=TFIDF\left(vt_{i},SGC_{Coll}\right)-TFIDF\left(vt_{j},SGC_{Coll}\right). \end{array}$$

By employing this, the relevance of each SGC’s dictionary term within the collection can be calculated, and terms with relevance zero (i.e., terms that occur in all SGCs and hence have no impact on MMIR) can be eliminated from each SGC. Additionally, the introduction of thresholds can further optimize the MMIR application. This means that not only are terms with relevance zero removed, but also, e.g., the most irrelevant 10% of terms. However, this calculation requires a re-processing of all SGCs of a collection, when new assets are added to the collection. To avoid this, the eliminated (i.e., nonrelevant) terms can be represented by a collection’s stop word $SGC_{STOP}$.

The introduction of this $SGC_{STOP}$ finally empowers the modeling of the Aboutness of a collection of Semantic Graph Codes with the metric $M_{ABT}$. By utilizing the abovementioned threshold, the size of $SGC_{STOP}$ can be increased and thus the relevance of the remaining collection. However, in many cases, a user- or application-driven definition of relevant feature vocabulary terms is required. This can be achieved by editing the calculated $SGC_{STOP}$ and extending it with additional feature vocabulary terms to produce an edited $SGC_{STOP-ED}$. The Aboutness of $SGC_{Coll}$ can now be defined as the distance of a SGC to $SGC_{STOP-ED}$ based on the original Graph Code metrics $M_{GC}=\left(M_{F},M_{FR},M_{RT}\right)$:

(16) $$\begin{array}{c} M_{ABT}=SGC-SGC_{STOP-ED}. \end{array}$$

As the three metrics in the relevance weighting and feature discrimination are now defined, applications can employ these metrics in a pre-processing step by filtering only relevant information, or by weighting these terms according to the MMIR application use cases. If, for example, the application scope is mammography, the information about broken legs might not be so relevant (calculated by applying $M_{REL}$). This Weighting and Filtering can also be regarded as Application Profiling for a given application context defined by $M_{ABT}$. Depending on $M_{REL}$ and the overall feature relevance, certain terms can gain particular interest for diagnoses. Based on $M_{DIS}$, the concept of emerging named entities [45] can also be improved.

**Example** **7.** *If the term “COVID-19” is mentioned frequently in a doctor’s letter, the corresponding $M_{DIS}$ value will increase and thus indicate that this particular letter has “COVID-19” as a highly relevant feature vocabulary term.*

#### 3.2.5. Feature Relevant Semantic Graph Codes

Whenever a new *SGC* is calculated within the collection, the collection’s $SGC_{STOP}$ is subtracted from it (i.e., any dictionary term including all its relationships is removed from the first *SGC*, if it exists in $SGC_{STOP}$), resulting in a compressed and Feature Relevant Semantic Graph Code FRSGC:

(17) $$\begin{array}{c} FRSGC=SGC-SGC_{STOP}. \end{array}$$

The following list shows some typical use cases for this FRGCs in the medical application domain:

- $FRSGC_{P}\left(i\right)$: basically, each patient’s Graph Code $GC_{P}\left(i\right)$ will contain lots of similar information, e.g., name, date of birth, and gender, and will be included in any contributing MMIR asset. However, this information is not relevant for MMIR processing and would be removed by the calculation of $FRSGC_{P}\left(i\right)$.
- $FRSGC_{App}$: medical or diagnostic applications typically do not need any existing MMIR feature for their internal processing. A hematologist, for example, would not be particularly interested in a broken knee, but be highly interested in the history of blood parameter measures. In this case, for the calculation of $FRGC_{App}$ the whole collection of $GC_{P}$ (i.e., the Graph Codes of all patients) will be employed as a baseline for the calculation of the TFIDF measure. As all patients will have blood parameter measures, and only a few will have broken knees, the latter information will be regarded as irrelevant.
- $FRSGC_{Hist}$: due to the timed nature of $GC_{P}$, emerging arguments can also be calculated based on FRSGCs. For example, beginning with the COVID-19 crisis in 2020, terms such as “PCR test”, ”vaccinated”, or “Corona-positive” were introduced in many medical documents and, of course, became highly important information. A $FRSGC_{Hist}$ is based on the concept of emerging named entities [45] and calculated on the union of intersections of $GC_{P}$ in a medical collection. This means that only the differences in a patient’s GC compared to previous versions are regarded as being relevant (e.g., when the patient took his first COVID-19 test), and then the union of all the patient’s differences in a certain period is constructed as a baseline for TFIDF. Such a calculation clearly indicates that terms such as “COVID-19” have become more relevant since 2020.

Formally speaking, for a collection of *n* patients in a period of time from t1−t2, this can be summarized as:

(18) $$\begin{array}{c} FRSGC_{Pi}=TFIDF\left(\overset{t}{\underset{i=0}{⋃}}SGC_{Pi}\right) \end{array}$$

(19) $$\begin{array}{c} FRSGC_{App}=TFIDF\left(\overset{n}{\underset{i=0}{⋃}}SGC_{Pi}\right) \end{array}$$

(20) $$\begin{array}{c} FRSGC_{Hist}=TFIDF\left(\overset{n}{\underset{i=0}{⋃}}\overset{t2}{\underset{j=t1}{⋂}}SGC_{Pj}\right). \end{array}$$

Particularly, $FRGC_{Hist}$ can be regarded as a sound basis for subsequent data mining or prediction processes. Depending on the application purpose, combinations of $FRSGC_{P}\left(i\right),\;FRGC_{App}$, and $FRGC_{Hist}$ are also possible. Feature Relevant Semantic Graph Codes can also be employed as a basis for further semantic modeling, as discussed in the next section. With the definition of these metrics, the open challenge in the area of MMIR, i.e., the semantic representation of features and the calculation of their relevance, has been addressed. To achieve the second goal of Explainability, a further step is required, which introduces a semantic understanding of true and false information.

### 3.3. Intelligent Information Retrieval, Reasoning, and Inferencing

The previously defined Feature Relevant Semantic Graph Codes can now be employed for inference calculation and reasoning to particularly support medical diagnosis systems. In general, inferencing and reasoning require the categorization of information in terms of valid or invalid. Such information can be maintained in Knowledge Organization Systems (KOSs) or Truth Maintenance Systems [28,37]. However, in many cases, such systems are not available. Hence, in the following subsection, an approach based on Feature Relevant Semantic Graph Codes is taken to support the calculation and maintenance of validity for an MMIR collection. This modeling is aligned with the concept of nonmonotonic reasoning [29], based on justified beliefs and reasonable assumptions. Furthermore, a Default Logic according to Poole [30,31] is applied. As shown in Section 2, such a Default Theory

(21) $$\begin{array}{c} T_{Poole}=\left(F,D\right) \end{array}$$

is described by the set F of facts (i.e., justified beliefs) and the set D of hypotheses (i.e., possible assumptions). The overall goal is to increase the number of elements in F by applying rules or calculations to the elements in D (i.e., building Knowledge Extensions). This is achieved by defining a measure for the Knowledge Validity KV of a collection based on the feature relevant relationship types between feature vocabulary terms, which are measured by the metric $M_{RT}$ as follows:

(22) $$\begin{array}{c} KV\left(vt_{i},vt_{j}\right)=log\frac{M_{RT}\left(FRSGC_{Coll}\left(vt_{i},vt_{j}\right)\right)}{|FRSGC_{Coll}\left(vt_{i},vt_{j}\right)|}. \end{array}$$

This formula calculates the ratio between similar relationships and possible relationships of vocabulary terms according to their relevance within the collection. The more similar the relationships that are detected, the higher the probability that this relationship is part of F and can be regarded as valid for the collection. Additionally, the introduction of a threshold (see Section 3.2) (e.g., a validity of say 90%) can lead to a significant increase in F and accurate approximations for inference. Based on the set F, automated reasoning falls to simple Boolean algebra tasks, as only relevant information is now contained in F. For this task, existing tools such as Apache Jena [46] can be integrated as a simple rule engine. The knowledge validity KV introduces automated reasoning and inference calculation for any Semantic Graph Code-based MMIR application. Paired with the additional concepts of this section, and according to [47], the GMAF, MMFG, and Graph Code retrieval can now be regarded as Intelligent IR.

**Example** **8.** *If, in all collection’s images of a person, the spatial relationship “head above person” has been detected, the feature relationship “above” between the vocabulary terms “head” and “person” can be regarded as common knowledge within the collection and will be added to the set F. If all images with a “hat” have the feature relationship “above” to the vocabulary term “head”, this information will also be added to F. A re-calculation of the set of possible hypotheses D can now verify that the hypothesis “hat above person” is also true and move it to the set F.*

Until now, internal IR processing has mainly been addressed by the modeling of this section, which is typically hidden from users. However, the presentation and particularly the Explanation of MMIR processes are important in the medical application area. Therefore, in the next section, the presentation of explaining texts for different user groups is shown.

### 3.4. Explainability for Graph Codes

In this section, we discuss our approach to answering questions such as, e.g., “what happened between January and July?”, or “why is this element in the result list?”, in a human-understandable way. As our goal is to produce natural language sentences based on index features (i.e., based on Graph Codes), we will employ the grammar for correct English sentences $G_{en}$ from Section 2 as a basis. A typical use case in the area of medical applications and diagnosis is the detection or calculation of differences between two dates $t_{1}$, $t_{2}$ and different corresponding sets of patient data $GC_{P}\left(t_{1}\right)$ and $GC_{P}\left(t_{2}\right)$. The calculation of a $GC_{P-Diff}$ that was already discussed leads to a Graph Code, which only contains the differences between $t_{1}$ and $t_{2}$ and thus the information set to answer the questions above.

This modeling in general follows the Explainability of MMFGs as defined in [1] and hence introduces production rules for the English language, which are based on syntactic elements of a Graph Code, i.e., the dictionary terms, and the corresponding relationships. However, as Graph Codes are highly optimized 2D indexing structures, producing sentences falls to the task of defining production rules for certain MMIR use cases. For highly sophisticated explanations of MMIR assets, ESMMFGs can be employed (see [1]). Instead, Graph Codes can be employed for the explainability of MMIR processing steps. To calculate natural language texts based on $GC_{P-Diff}$, the dictionary terms $dict_{P-Diff}$ (i.e., the textual representation of the MMIR feature) of $GC_{P-Diff}$ are mapped by the introduction of an LBL element to existing English language terms. The relationships between these dictionary terms (i.e., the value of matrix cell $m_{i,j}$) can also be formulated in natural language. This can be achieved by assigning each relationship type of a Graph Code (i.e., each possible value of a matrix cell $m_{i,j}$) a pre-defined phrase $PHR_{type}$. For example, $PHR_{4}$ could be represented by the phrase “is a”, $PHR_{3}$ could be represented by “belongs to”. The calculation of which data are actually in the Graph Code depends on the MMIR processing step. There are two general use cases for explainability and corresponding Graph Code calculations:

- “Why is this element in the result list?”. This question can be answered based on *FRSGCs* by calculating the subtraction of the result element $FRSGC_{i}$ with the original query object, which can represented by an $FRSGC_{Query}$. In this case, an Explaining Semantic Graph Code (ESGC) would be $ESGC=FRSG_{i}-FRSGC_{Query}$.
- “Why is this element before the other?”, or “what is the difference between these elements?”. In this case, the ESGC would be calculated by the subtraction of the two result elements $FRSGC_{i},\;FRSGC_{j}$ as $ESGC=FRSGC_{i}-FRSGC_{j}$.

We can now introduce the grammar $G_{ESGC}=\left(V_{en},T_{en}\cup LBL,P_{SGC},S_{en}\right)$ for Graph Codes by the definition of the following production rules $P_{GC}$ to formally produce natural language sentences for the explanation of search results:

(23) $$\begin{array}{c} P_{GC}=P_{en}\cup \{ \end{array}$$

(24) $$\begin{array}{c} dict_{answer}\to LBL \end{array}$$

(25) $$\begin{array}{c} m_{i,j}\neq 0\to LBL\;\;PHR_{type}\;\;LBL \end{array}$$

(26) $$\begin{array}{c} \}. \end{array}$$

When this grammar is employed for Semantic Graph Codes, further information from internal or external SKOSs can be used for the calculation of human-understandable explanations. When this grammar is employed for Feature Relevant Graph Codes, the resulting explanations become parsimonious and accurate, while the explanation of a regular Graph Code will contain more (and possibly superfluous) information. However, in any case, the production rules remain the same. Based on these rules, any processed Graph Code becomes a Explainable Semantic Graph Code (ESGC). Based on $G_{GC}$, the following explanation can be calculated for a given $GC_{P-Diff}$: “between January and July, the blood pressure increased from 140 to 155”.

As these phrases are formally correct English sentences, and are based on the complete set of English grammar rules, further language generation optimizations can be applied by adapting the phrases $PHR_{type}$ and the production rules $P_{SGC}$. A basic implementation of such rules has been completed in the context of the GMAF framework and will be discussed in the next section.

## 4. Implementation and Evaluation

For the implementation of this modeling, we added several GMAF plugins, FRGSCs, and ESGCs to the GMAF framework. Here, we only discuss the final result from a user’s perspective. Hence, here feature relevance and corresponding results are demonstrated, and details on the implementation of explainability will be given. The current version of the GMAF and the corresponding implementations are available on Github [48]. This evaluation is based on a detailed quantitative and qualitative experiment, which has been published in previous work [2]. Here, we focus on the extension of our work and the corresponding results in the medical context.

To demonstrate modeling and implementation results, in this section two classes of datasets have been employed: (1) one very general dataset with MMIR assets from the nonmedical context to provide a simple and intuitive understanding of the experiment and the interpretation of the corresponding result, and (2) a second dataset from the medical context to show that the presented approach can easily be transferred to other application domains, that the results confirm the hypothesis, and that Graph Codes can improve MMIR for the medical application domain. In Section 4.1, feature relevance and the corresponding metrics are demonstrated, and Section 4.2 contains experiments in the area of explainability.

### 4.1. Demonstration of Feature Relevance

**Figure 6:**
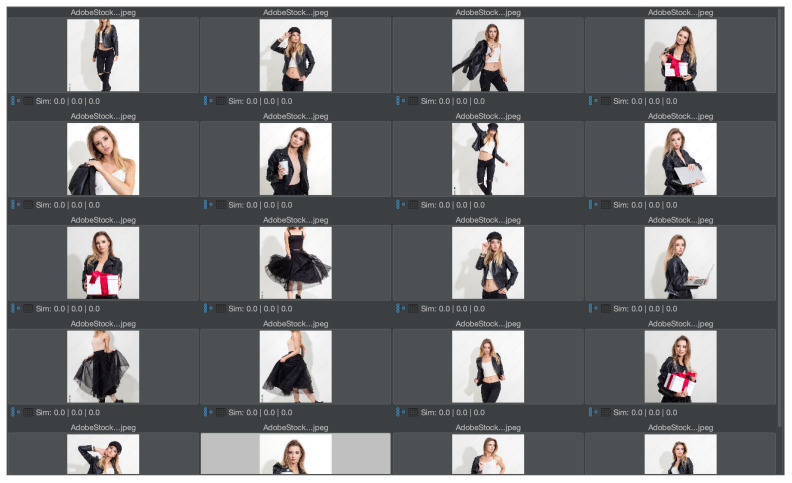
Picture collection from a photo shoot shown in the GMAF framework.

**Figure 7:**
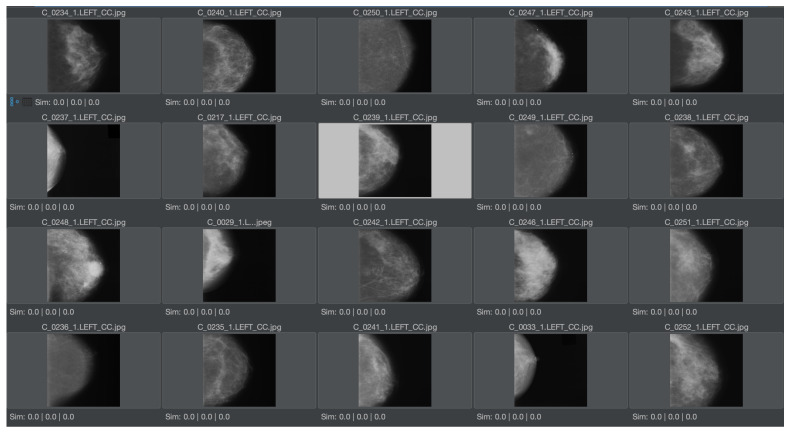
Picture collection of mammography images shown in the GMAF framework.

**Figure 8:**
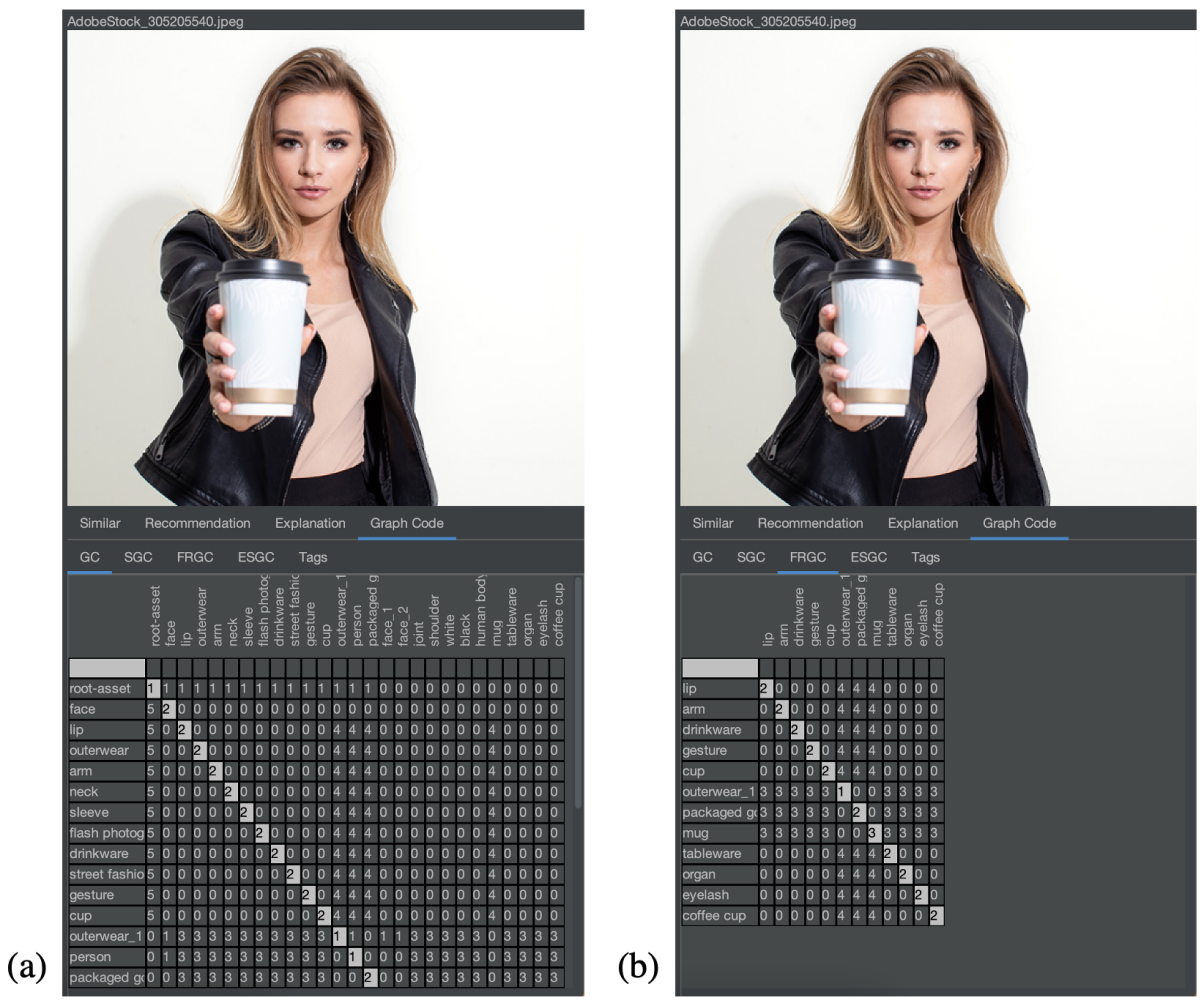
Comparison of (**a**) Graph Code and (**b**) Feature Relevant Graph Code.

**Figure 9:**
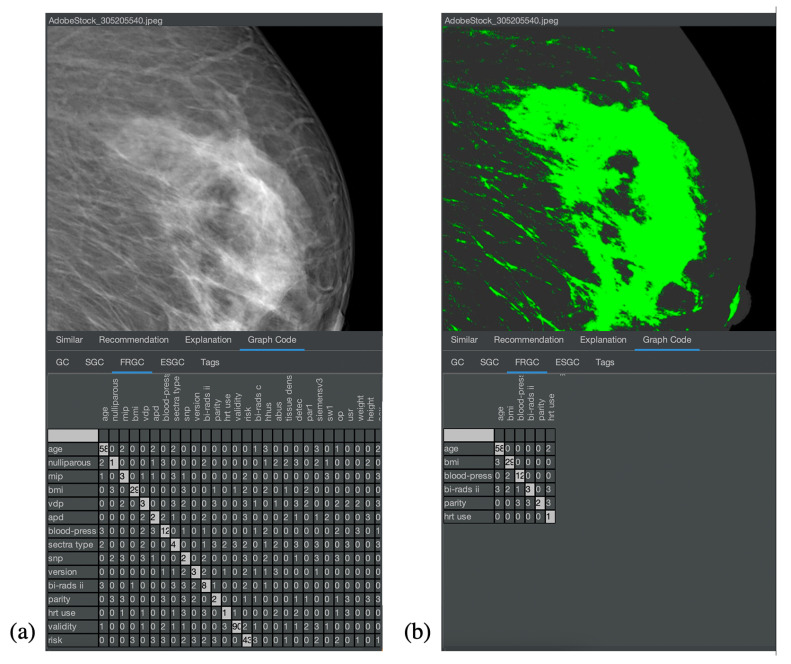
Comparison of (**a**) Graph Code and (**b**) Feature Relevant Graph Code in the medical application domain.

The first experiment shown here is general in order to demonstrate feature relevance. The second experiment shows the same task in a medical context. In particular, the first experiment should provide an intuitive understanding of the solution for users without a medical background. Hence, a test collection of mainly similar pictures has been created. The images are licensed from Adobe Stock and illustrate a photo shoot context with a model, where many pictures with a similar setup have been taken (see Figure 6). In Figure 7, a similar collection in the medical domain is shown, which serves as a dataset to demonstrate the same experiment in a medical context. The pictures are taken from [12], which is a publicly available and anonymized dataset for medical image features. As all of these pictures have numerous common elements for MMIR, it would be important to find differences, i.e., the relevant features of each picture in the context of the collection. Hence, the Feature Relevant Graph Code has been introduced (see Section 3.2) and should be validated in this experiment. Figure 8 and Figure 9 show the corresponding result for the nonmedical and medical contexts. The original images are displayed at the top of Figure 8a,b and their respective Graph Code visualization is shown at the bottom. Figure 9 is similar. In Figure 8a and Figure 9a, the normal Graph Code is shown, which contains all the vocabulary terms detected by the MMIR processing of this image. Many of them are similar in most pictures in the collection and hence irrelevant for MMIR. The Feature Relevant Graph Code (FRGC) in Figure 8b and Figure 9b, however, only contains such vocabulary terms that are unique for the selected picture and hence highly relevant for MMIR. Furthermore, the FRGC leads to additional compression. In Figure 8, only 12 relevant features are contained in the Graph Code instead of 36 before the relevance calculation. The number of matrix fields to be processed is hence reduced from 1296 to 144, which is a reduction of 88%. The resulting Feature Relevant Graph Codes in the medical context (see Figure 9) show a similar compression rate, i.e., the detection of relevant features increases significantly the quality of the medical collection’s index.

This experiment shows clearly that relevance calculations based on Graph Codes and the TFIDF algorithm lead to a compression of the index and also to highly relevant individual features in the medical context. In the next section, these Feature Relevant Semantic Graph Codes are employed for the introduction of explainability.

### 4.2. Demonstration of Explainability

**Figure 10:**
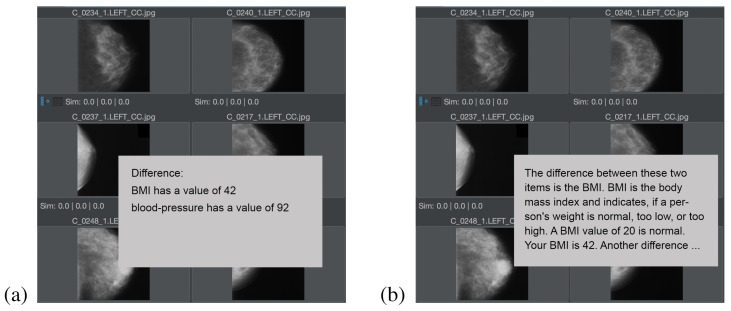
Explaining tooltip of a result element for (**a**) a medical target audience and (**b**) a nonmedical target audience (**b**).

For the implementation of explainability, we decided to present a minor mouse-over tooltip to the user during mouse hover over an item in the result area. This tooltip contains the basic natural language explanation as to why this element is part of the result list. The definition of detailed asset descriptions is based on MMFGs (and not the Graph Code index structure) and has already been introduced and demonstrated [1]. Figure 10 shows the explanation of the question “what is the difference between two elements?”, in the medical context for two target audiences, whereas in Figure 10a an explanation for a medical target audience (i.e., doctors or nurses) is calculated, and in Figure 10b, an explanation for a nonmedical audience (i.e., the patient) is calculated. In both cases, the ESGC contains only a few vocabulary terms, which is typical for the difference calculation (i.e., the subtraction) of two feature relevant elements. In the case of this example, the ESGC only contains the vocabulary terms “BMI” (i.e., the body mass index) and “blood-pressure”.

For the medical audience, short phrases $PHR_{type}$ for the explanation of ESGC have been selected, e.g., LBL is the value of the vocabulary term, $PHR_{3}=$ has a value of. For the nonmedical audience, the LBL objects have been defined in a way that, e.g., the LBL for the term BMI is not just “BMI”, but “BMI is the body mass index and indicates if a person’s weight is normal, too low, or too high. A BMI value of 20 is normal”. $PHR_{3}$ for the nonmedical audience is defined as $PHR_{3}=$ Your value is.

This demonstration supports our hypothesis that automatically calculated explanation texts can improve the MMIR experience for any application domain, but particularly address requirements in the medical area, where different explaining texts for different target audiences are helpful.

### 4.3. Evaluation Planning

In order to perform a first evaluation of these results in the medical application area, a cognitive walkthrough with users (i.e., doctors, nurses) in the medical domain is planned following the methodology of [49]. For this evaluation, we plan the following tasks:

- Work task analysis: define the typical tasks in a real-world scenario in the medical application area, supported by results demonstrated in this paper.
- Walkthrough: evaluate if the demonstrated solution can increase efficiency and/or effectiveness of the work task, including a detailed discussion and documentation of benefits, errors, and improvements.
- Questionnaire: after the walkthrough, the users are asked to provide feedback in the form of a questionnaire, which will allow further statistical evaluations.

The planning of such an evaluation is currently ongoing. The evaluation and respective results of this evaluation are beyond the scope of this paper.

## 5. Conclusions and Future Work

In this paper, the transfer and extension of Graph Codes for fast and effective MMIR in the medical application domain have been discussed. In particular, for medical applications, the calculation of relevant information is important due to the significant number of MMIR assets that are produced during diagnosis and treatment. We showed that Relevance Metrics can be defined and employed for calculations on a semantic feature level if certain MMIR assets or vocabulary terms are relevant for the application use case. In the medical context, this helps doctors and nurses to focus on their primary task and minimize time-intensive pre-filtering of information. Furthermore, these metrics enable application profiling for special application use cases, which further increases the improvement of Graph Codes in the medical application domain. The definition of Discriminating features and the corresponding value ranges can be employed to introduce thresholds or limits for certain feature values, which can be further employed for human-understandable explanations of medical features and their values. These explanations are introduced based on a PS-Grammar, which can be employed to generate automated texts for different target audiences. This modeling hence answers the research question and provides a solid foundation for further applications of Graph Codes in the medical application domain.

However, the approach presented is not just applicable for the medical application domain. In fact, the modeling and implementation are general and application domain agnostic and can be employed in any MMIR domain. Hence, transfer to the medical application domain can be regarded as a blueprint for transferring Graph Code-based MMIR to other application domains. This can form part of our future work.

## Figures and Tables

**Table 1:** Exemplary Graph Code for the biometrical landmark “Eye Distance”, where distance values (i.e., rows and columns labeled “27.21” and “18.14”) are stored as vocabulary terms.

*GC*	Face 1	Face 2	Eye Distance	27.21	18.14
Face 1	1	0	0	0	0
Face 2	0	1	3	0	0
Eye Distance	3	3	1	0	0
27.21	3	0	3	1	0
18.14	0	3	3	0	1

**Table 2:** Exemplary Graph Code with discrete values, where distance values are stored as matrix cell values of the row and/or column named “Eye Distance”.

*GC*	Face 1	Face 2	Eye Distance
Face 1	1	0	D(27.21)
Face 2	0	1	D(18.14)
Eye Distance	0	0	1

**Table 3:** Exemplary mapping of database table rows and columns into Graph Codes.

${\mathrm{GC}}_{{\mathrm{DB}}_{R}\mathrm{ow}}$	Firstname	Lastname	Street	Zipcode
firstname	*D* (*John*)	0	0	0
lastname	0	*D* (*Doe*)	0	0
street	0	0	*D* (*Nowhere Road*)	0
zipcode	0	0	0	*D* (*1234*)

## Data Availability

The data presented in this study are openly available in [48].

## References

[1] Wagenpfeil S., McKevitt P., Hemmje M. (2021). Towards Automated Semantic Explainability of Multimedia Feature Graphs. Information.

[2] Wagenpfeil S., McKevitt P., Hemmje M. (2021). Fast and Effective Retrieval for Large Multimedia Collections. Big Data Cogn. Comput..

[3] Wagenpfeil S., MvKevitt P., Hemmje M. (2021). AI-Based Semantic Multimedia Indexing and Retrieval for Social Media on Smartphones. Information.

[4] Hemmje M., Jordan B., Pfenninger M., Madsen A., Murtagh F., Kramer M., Bouquett P., McIvor T. (2020). Artificial Intelligence for Hospitals, Healthcare & Humanity (AI4H3).

[5] Cheddad A., Czene K., Shepherd J., Li J., Hall P., Humphreys K. (2014). Enhancement of mammographic density measures in breast cancer risk prediction. Cancer Epidemiol. Biomarkers Prev. (CEBP).

[6] Cheddad A., Czene K., Eriksson M., Li J., Easton D., Hall P., Humphreys K. (2014). Area and volumetric density estimation in processed full-field digital mammograms for risk assessment of breast cancer. PLoS ONE.

[7] Strand F., Humphreys K., Cheddad A., Törnberg S., Azavedo E., Shepherd J., Hall P., Czene K. (2016). Novel mammographic image features differentiate between interval and screen-detected breast cancer: A case-case study. Breast Cancer Res..

[8] Holtzmann-Kevles B. (1997). Naked to the Bone: Medical Imaging in the Twentieth Century.

[9] Slichter P. (2013). Principles of Magnetic Resonance.

[10] Seeram E. (2013). Computed Tomography-E-Book: Physical Principles, Clinical Applications, and Quality Control.

[11] Dornheim Segmenter.com (2020). DICOM Viewer. https://www.dornheim-segmenter.com/en/products/dicom-viewer-free/.

[12] Lekamlage C.D., Afzal F., Westerberg E., Cheddad A. Mini-DDSM: Mammography-based Automatic Age Estimation. Proceedings of the 3rd International Conference on Digital Medicine and Image Processing (DMIP 2020).

[13] Ichalkaranje N. (2006). Intelligent Paradigms for Assistive and Preventive Healthcare.

[14] Nunamaker J., Chen M., Purdin T.D.M. (1991). Systems Development in InformationSystems Research. J. Manag. Inf. Syst..

[15] Aberer K., Choi K., Noy N., Allemang D., Lee K., Nixon L., Goldbeck J., Mika P., Maynard D., Mozoguchi R. The Semantic Web.

[16] Subrahmanian V.S. (1998). Principles of Multimedia Databases.

[17] Nixon M. (2020). Feature Extraction and Image Processing for Computer Vision.

[18] Bhute A. (2012). Multimedia Indexing and Retrieval Techniques: A Review. Int. J. Comput. Appl..

[19] Beyerer C. (2017). Pattern Recognition—Introduction.

[20] Kurland O. Fusion in Information Retrieval: SIGIR 2018 Half-Day Tutorial. Proceedings of the 41st International ACM SIGIR Conference on Research & Development in Information Retrieval.

[21] Leveling J. Interpretation of coordinations, compound generation, and result fusion for query variants. Proceedings of the 36th International ACM SIGIR Conference on Research and Development in Information Retrieval.

[22] Lew M. (2006). Content-Based Multimedia Information Retrieval: State of the Art and Challenges. ACM Trans. Multimed. Comput. Commun. Appl. (TOMM).

[23] Hernandez C. Data Fusion and Label Weighting for Image Retrieval Based on Spatio-Conceptual Information. Proceedings of the Adaptivity, Personalization and Fusion of Heterogeneous Information, RIAO ’10.

[24] Dufour R. Local and global models for spontaneous speech segment detection and characterization. Proceedings of the 2009 IEEE Workshop on Automatic Speech Recognition & Understanding.

[25] Chang S.-F. (2001). Overview of the MPEG-7 standard. IEEE Trans. Circuits Syst. Video Technol..

[26] FFMpeg.org (2020). FFMPEG Documentation. http://ffmpeg.org.

[27] Mu X. Content-Based Video Retrieval: Does Video’s Semantic Visual Feature Matter?. Proceedings of the 29th Annual International ACM SIGIR Conference on Research and Development in Information Retrieval.

[28] Beierle C. (2019). Methoden Wissensbasierter Systeme—Grundlagen.

[29] Bochman A. (2005). Nonmonotonic Reasoning.

[30] Poole D. (1988). A logical framework for default reasoning. Artif. Intell..

[31] Das A. (2003). Knowledge Representation. https://www.sciencedirect.com/science/article/pii/B0122272404001027.

[32] Hawke P. (2018). Theories of Aboutness. Australas. J. Philos..

[33] Wagenpfeil S., McKevitt P., Hemmje M. (2021). Graph Codes-2D Projections of Multimedia Feature Graphs for Fast and Effective Retrieval. Int. J. Comput. Inf. Eng..

[34] Sciencedirect.com (2020). Adjacency Matrix. https://www.sciencedirect.com/topics/mathematics/adjacency-matrix.

[35] Asim M.N., Wasim M., Khan M.U.G., Mahmood N., Mahmood W. (2019). The Use of Ontology in Retrieval: A Study on Textual. IEEE Access.

[36] Domingue J. (2011). Introduction to the Semantic Web Technologies.

[37] W3C (2021). SKOS Simple Knowledge Organisation System. https://www.w3.org/2004/02/skos/.

[38] Aho A. (1998). Compilerbau.

[39] Hauser R. (2000). Principles of Computer Linguistics.

[40] Larobina M. (2014). Medical Image File Formats. J. Digit. Imaging.

[41] Norman S. (1985). User Centered System Design—New Perspectives on Human-Computer Interaction.

[42] Silge J., Robinson D. (2022). Text Mining with R: A Tidy Approach.

[43] Eljasik-Swoboda T. (2020). Explainable and Transferrable Text Categorization.

[44] Eljasik-Swoboda T. No Target Function Classifier: Fast Unsupervised Text Categorization Using Semantic Spaces. Proceedings of the 7th International Conference on Data Science, Technology and Applications-DATA.

[45] Duttenhöfer A., Wagenpfeil S., Hemmje M. Supporting Argument Strength by Integrating Semantic Multimedia Feature Detection with Emerging Argument Extraction. Proceedings of the Argstrength Workshop.

[46] Apache Software Foundation (2020). Reasoners and Rule Engines: Jena Inference Support. https://jena.apache.org/documentation/inference/.

[47] Belkin N.J. (1996). Intelligent Information Retrieval: Whose Intelligence?. Ingénierie Des Systèmes D’information-ISI.

[48] Wagenpfeil S. (2021). Github Repository of GMAF and MMFVG. https://github.com/stefanwagenpfeil/GMAF/.

[49] Spencer R. The streamlined cognitive walkthrough method, working around social constraints encountered in a software development company. Proceedings of the SIGCHI Conference on Human Factors in Computing Systems (CHI ’00).

